# Methods considerations for nystagmography

**DOI:** 10.1186/s40463-015-0078-2

**Published:** 2015-06-24

**Authors:** Brian W. Blakley, Laura Chan

**Affiliations:** Department of Otolaryngology, University of Manitoba, GB420-820 Sherbrook Street, Winnipeg, MB R3A 1RJ Canada

## Abstract

**Objectives:**

1. To assess the reproducibility of eye movement velocity measurement using two methods: traditional electro-oculography (EOG) and infrared video-oculography (VOG) and,

2. Determine whether the normal values for unilateral weakness and bilateral reduction of caloric responses vary according to method employed.

**Background:**

Vestibular testing frequently involves measurement of eye movements. EOG has been the standard method for decades, but VOG and other methods have recently become popular. The assumption has been that all methods measure eye movements equally and accurately but this assumption has not been validated. In this paper we examine this assumption.

**Methods:**

Eye movements were recorded simultaneously with commercially available EOG and VOG methods to evaluate differences in results for nineteen normal subjects undergoing caloric tests with warm and cold water. Examination of the records permitted identification and simultaneous measurement of 840 nystagmus beats.

**Results:**

EOG and VOG measurements were correlated but the correlation was not strong (Spearman rho = 0.529, p < 0.01). Eye velocities recorded by the VOG system were greater than that for the EOG system. The mean VOG/EOG ratio was 1.71. Normal values used at our centre were adjusted to accommodate the use of video technology to account for the differences in sensitivity between EOG and VOG methods.

**Conclusion:**

The traditional EOG-based normal value for bilateral reduction of caloric response, 30 degree per second (d/s) based on traditional EOG measurements should be revised to 50 d/s for modern VOG testing in our lab. Normal values for vestibular testing may need to be re-evaluated when new technology is introduced. Each lab should verify normal values for their own methods and equipment.

## Introduction

New clinical testing techniques should be accompanied by re-evaluation of normal values. Recently developed eye movement measurement techniques include infrared, video technology and scleral search coil technology [1–3]. Of these, the scleral search coil technique is generally agreed to be the most accurate, but its use requires the patient to wear a contact lens and the equipment is expensive and prone to problems [2, 4].

The goal of this report is to assess the concurrence between electro-oculography (EOG) and video-oculography (VOG) measurements. For decades, the electrical difference between the cornea and the retina of the eye has been used to record eye movements. This electrical potential difference is small but with amplification and proper filtering it can be detected using surface electrodes. This is called electro-oculography. When EOG is used to record eye movements for caloric, saccade, pursuit and other tests, it is part of the electronystagmography (ENG) battery of tests. If video techniques are used, the test may be called videonystagmography and the eye movement recordings are video-oculography (VOG). EOG and/or VOG can be used to measure the response to caloric stimulation and is called nystagmography [5]. Some fundamental differences between EOG and VOG that could be clinically important have become apparent after experience and consideration of the recording technique [5–7]. Some of these are indicated in Table 1.

**Table 1:** Some differences between EOG and VOG

	ELECTRO-oculography (EOG)	VIDEO-oculography (VOG)
Entity measured	Corneo-retinal electrical potential	Digitized position of a black circle presumed to be the pupil
Drift	Shift of baseline if DC recording used.	Theoretically no shift
	Variable if AC recording	
Artifact	Eye blinks and muscle contraction are the most frequent artifacts	Dark features such as mascara, closed eyes, eye brows “fool” the system momentarily.
		Eye blinks, difficulty detecting the pupil causes large artifacts
Sampling rates	While most commercial units sample calorics at 30 Hz, much higher sampling rates are feasible. This is critical for accurate measurement of quick phases	Video sampling rates are usually 30–60 Hz. Sampling rates of 100 Hz requires specialized equipment.
Ease of use	Sticky electrodes are required with possible impedance problems, electrical drift and small signal	The patient wears goggles to mount the camera to, which limits eye displacement to approximately 20°
Determination of maximum slow phase velocity (SPEV)	Maximum average SPEV of the three greatest consecutive beats	Maximum average SPEV for a 10 s window of recording

Normal values for clinical caloric testing have been based on traditional EOG measurements. The most important parameter is the unilateral weakness (UW) [3, 5, 6] which assesses the percent difference of the maximum slow phase velocity (SPEV) for warm and cool water stimuli in each ear. UW may be called reduced vestibular response or caloric weakness but is the same measure. Values are considered abnormal for clinical purposes if the UW is greater than 25 % or the sum of the four caloric tests (right warm + right cool + left warm + left cool) is less than 30 d/s [5, 6]. We conducted this study to evaluate whether EOG and VOG methods provide equivalent results or whether some change in the values scale is needed according to the technique used.

Several methods of recording eye movements have emerged so it is important to compare established technique with newer ones as they are brought into practice. Fortunately, it is possible to use both the EOG and VOG systems at the same time to study exactly the same eye movements. As far as we are aware, this is the first paper in the literature to directly compare EOG and VOG results simultaneously recorded.

The number of ways that eye movement measurement techniques can be employed is large. Computer algorithms and their underlying assumptions, sampling rates, filtering, DC electrical shift, noise, and other factors may produce different results but consideration of the effects of different methods are not prominent in the literature [8, 9]. This project was performed using commercially available equipment that is assumed to represent valid general protocols for electro- and video- techniques. In deference to the possible sensitivity that manufacturers may have, the names of the companies are not included and are not considered important. Differences between the two techniques, EOG and VOG, are the focus of this paper.

## Methods

We measured the slow phase velocity for the same nystagmus beats using both EOG and VOG techniques simultaneously. Preliminary data suggested that we could expect to identify at least ten simultaneous slow-phase eye movements in each subject. We wished to have results with statistical power greater than 95 %. Using SYSTAT 3.5’s power calculator and applying the standard deviation of 8 d/s and the mean difference of 3 d/s we calculated that 19 normal subjects would be required to detect a difference of 3 d/s with 95 % power at the *p* = 0.05 level. In fact, we were able to determine over 40 simultaneous SPEVs per subject.

The Human Research Ethics Committee of the University of Manitoba approved the protocol. Subjects were 19 normal volunteers who had no complaints of dizziness or ear dysfunction and who had normal otoscopic exams. EOG recording was carried out with electrocardiogram-type surface electrodes. These were applied posterior to the lateral canthus bilaterally, above and below the left eye and in the center of the forehead (reference electrode). Caloric irrigations were performed similar to ANSI standards [7] - the sampling rates were 30 Hz for EOG and 60 Hz for VOG caloric tests. Warm and cool water irrigations at 44 °C and 30 °C respectively for 20 s at 200 cm^3^/min were administered in each ear with eye movement recording for 60 s. VOG recording was carried out with a camera mounted in the right eye of specially designed goggles as part of a commercially available system. Each eye was recorded with a different technique. Conjugate eye movements were assumed. The EOG electrodes were worn under the goggles, which helped stabilize the electrodes. Both systems were calibrated according to the manufacturer’s directions using arrays of red diode lights. Examining the EOG and VOG records together allowed us to identify the two system’s measurement of the same beats of nystagmus. The velocities measured with both methods were entered into a database.

Statistical analysis: Eye velocity data departed from a normal distribution so non-parametric tests were applied. The correlation between EOG and VOG recordings was calculated using Spearman’s rho and the significance of differences assessed with the Wilcoxon signed ranks test. For UW scores, the Pearson correlation coefficient and paired t-tests were used because the UW data were normally distributed. Statistical calculations were performed using IBM SPSS v22.0.

The Jongkees formula [6] was used to calculate UW for the electro- and video recording systems for these normal subjects

$$ UW=\frac{\left(RW+RC\right)-\left(LW+LC\right)}{RW+RC+LW+LC} $$

where RW, RC, LW and LC refer to the responses for right ear warm, right ear cool, left ear warm, and left ear cool, respectively. If abnormal values were obtained for these normal subjects, doubt would be cast on the validity of the technique used.

The two parameters that are most significant clinically are UW and the sum of the four caloric tests. Abnormal UW suggests that responses from the two ears are not symmetric so one ear is hypo-responsive. If the sum of the four caloric tests is less than a normal threshold value, bilateral reduction of caloric response is present which suggests significant dysfunction. Even if the two systems provide different values for eye movements, and the error is linear, the UW should still be meaningful because UW is a ratio or relative measure. This is not true for the sum of the four caloric tests. The sum of the caloric tests is an absolute measure so it will vary according to the magnitude of the measures.

## Results

**Fig. 1:**
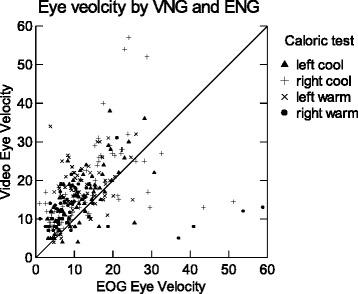
Eye velocities by EOG (abscissa) and VOG (ordinate). N = 840. If the correlation were perfect all the values would fall on the diagonal line. Many data points lie on top or nearly on top of each other. Velocities for VOG recording were higher than for the same beats of nystagmus recorded with EOG. The group of data points on the lower right of the figure suggests that large EOG velocities deviate much more from VOG than lower velocities. Spearman’s rho =0.529

**Fig. 2:**
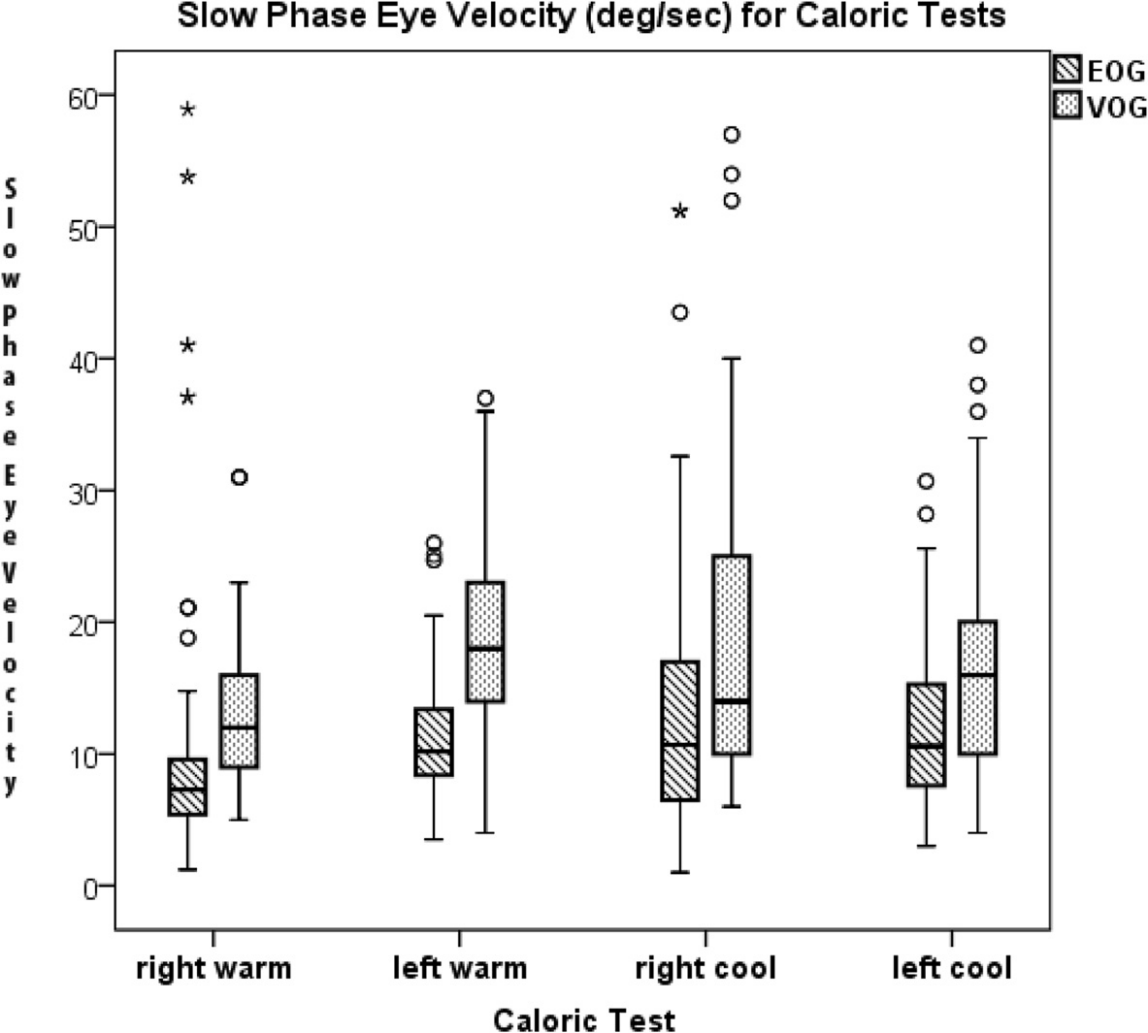
Box and whisker plot of SPEV showing the median (horizontal line), interquartile range (limits of the box), outliers (o), and extreme cases (*) of the four types of caloric tests. Over all caloric tests, the median SPEV was 9.6 and 16 for EOG and VOG, respectively. The EOG measure for the same SPEV was greater suggesting greater sensitivity of the EOG technique

Eight hundred forty slow phases from the resulting nystagmus were identified on both records according to the time that they occurred. Spearman’s rho for the measured velocity for EOG and VOG methods was only 0.529, departing significantly from perfect correlation of 1.0. EOG and VOG velocities were statistically significantly different (*p* < 0.001) and the correlation fell far short of our expectations, given that we were objectively measuring exactly the same quantity. Fig. 1 displays the bivariate relationship. Velocities for VOG recording were higher than for the same beats of nystagmus recorded with EOG. Fig. 2 illustrates the differences between EOG and VOG measures with box and whisker plots. The median velocities for VOG and EOG were 16 and 9.6 d/s respectively. The mean VOG/EOG ratio was 1.71.

Although the eye velocities were not normally distributed, the UW data were normally distributed so parametric tests were applied. The mean (+/−s.d.) unilateral weakness for EOG and VOG was 0.1 % (+/−22.3) and 9.3 % (+/−12.9) respectively. The Pearson correlation coefficient between unilateral weakness for EOG and VOG was 0.59, which is minimally better than the correlation for eye movement velocities.

**Fig. 3:**
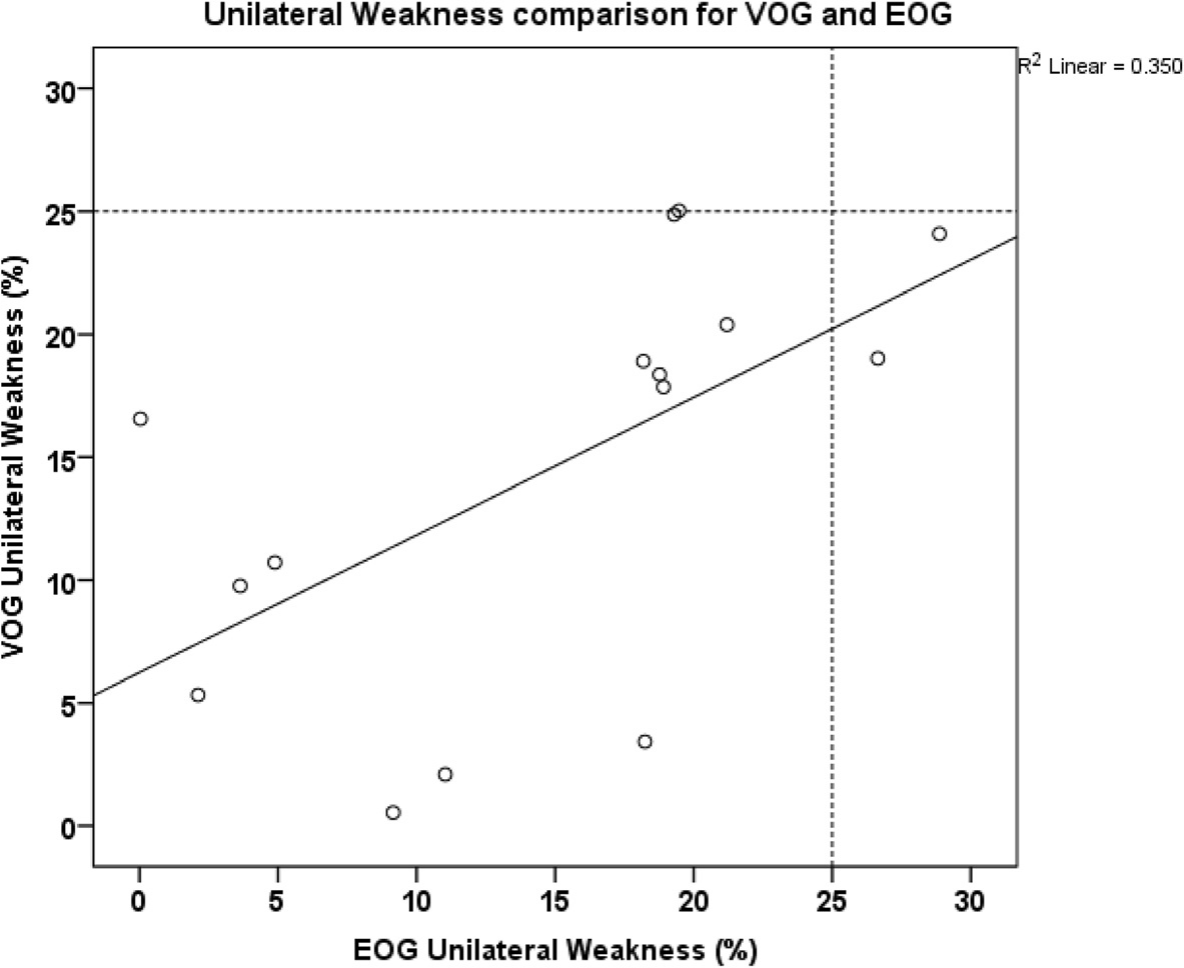
Unilateral Weakness as determined by video-oculography (VOG) *versus* electro-oculography (EOG) techniques. The R [2] value suggests that 35 % of the variability of the data is explained by the two variables which is less than we expected. We suspect that the variability of the EOG measurements accounts for much of the reduced R [2]. Note that at the accepted threshold for normal UW (<25 %), two normal subjects would have been incorrectly labeled as abnormal whereas none of the VOG measures suggested abnormal results

UWs for EOG and VOG (mean +/−s.d.) were 14.69 +/−8.7 and 14.46 +/−8.48. Although these means and standard deviations were close they were statistically significantly different using the paired *t*-test (*p* = 0.02). Note that at the accepted threshold for normal UW (<25 %), two normal subjects would have been incorrectly labeled as abnormal whereas none of the VOG measures suggested abnormal results as shown in Fig. 3.

## Discussion

For years, EOG measures have been based on the assumption that electrical potential difference between two surface electrodes is linearly dependent on degrees of rotation of the eye. This assumption must be violated because the change of corneoretinal potential occurs over a curved space but is detected between two points. As the eye displacement increases, the error under this assumption also increases. Even if the velocity measurements are inaccurate for EOG, they may still be clinically useful as long as the errors are made consistently. For these reasons, in the interest of quality, it seems important that each lab verify their own normal values using their own techniques and equipment.

New techniques of recording eye movements include scleral search coil techniques, infrared and video techniques. It is logical that the results from different techniques will vary to some extent but we were disappointed at how poorly EOG and VOG results correlated. EOG usually yielded lower velocities than VOG for the same slow phase eye movement. Based on these data, it appears that our normal values for the sum of four caloric tests should be reconsidered in our lab. The traditional upper limit of normal for the sum is 30 d/s using EOG methods. If VOG methods are used, this traditional normal limit should be multiplied by the ratio of the median magnitude of the velocities or 30 d/s × (16/9.6) = 50 d/s. For this reason 50 d/s is the normal value for our lab. Again, individual labs should verify these values.

For each technique, there is an infinite number of ways that the tests could be performed. Software development requires that certain assumptions be made about the data. These assumptions potentially affect results. Some of these include assumptions about optimal filtering, optimal digitization, and definition of slow phase, fast phase and artifact. The EOG system functioned properly but is old technology. While these assumptions may have accounted for some variability it seems likely that the nature of the technique (electro or video) was also a major source of error as suggested by the R [2] value of 0.35 in Fig. 3. The UW correlated a little better (R = 0.59) than absolute eye velocity measurements for the two methods. UW is a relative measure, so differences in eye velocity should give similar results assuming that measurements are accurate.

The finding that two normal subjects had an abnormal UW on EOG recording but not with VOG suggests that the lower velocities measured by EOG maybe more likely to give false results than VOG. This makes sense because variability in small velocities is more likely to result in apparent abnormal calculations for UW than larger velocities with smaller relative variability. For example, the sensitivity of EOG is approximately 2 d/s [6] or 21 % of the median EOG measure [6]. An error of the same magnitude would be only 12 % for VOG. All subjects in this study were asymptomatic normal subjects so we were reassured that UW measures were normal for VOG techniques. Since adopting the 50 d/s criteria for lower limit of normal for the sum of the four caloric tests, we have found that it seems reliable in explaining chronic, idiopathic imbalance in many patients. This will be the subject of another report on rotary chair testing.

This study has limitations. We could not assess all equipment by all manufacturers. It would also be interesting to see how the techniques differ across a range of abnormal subjects or those with eye problems.

## Conclusion

EOG and VOG techniques do not provide equivalent results. Normal values should be adjusted depending on technique used. When using video techniques, our upper limit for normal for the sum of the four caloric tests should be 50 d/s. VOG may provide more accurate caloric results but EOG can still be useful if normal values have been verified using consistent methods for the lab.

## References

[1] Gananca MM, Caovilla HH, Gananca FF (2010). Electronystagmography *versus* videonystagmography. Braz J Otorhinolaryngol.

[2] West PD, Sheppard ZA, King EV (2012). Comparison of techniques for identification of peripheral vestibular nystagmus. J Laryngol Otol.

[3] Szirmai A, Keller B (2013). Electronystagmographic analysis of caloric test parameters in vestibular disorders. Eur Arch Otorhinolaryngol.

[4] MacDougall HG, Weber KP, McGarvie LA, Halmagyi GM, Curthoys IS (2009). The video head impulse test: diagnostic accuracy in peripheral vestibulopathy. Neurology.

[5] Baloh RW, Honrubia V (2011). Nystgmography Clinical Neurophysiology of the Vestibular System 4th edition.

[6] Barber H, Stockwell C (1980). Caloric Test. Manual of Electronystagmography.

[7] ANSI (1999). Procedures for testing basic vestibular function Acoustical Society of America.

[8] Jalocha-Kaczka A, Pietkiewicz P, Zielinska-Blizniewska H, Milonski J, Olszewski J (2014). Sensitivity evaluation in air and water caloric stimulation of the vestibular organs using videonystagmography. Otolaryngol Pol.

[9] Bell SL, Barker F, Heselton H, MacKenzie E, Dewhurst D, Sanderson A (2014). A study of the relationship between the video head impulse test and air calorics. Eur Arch Otorhinolaryngo.

